# Supplementary material for: Efficient intervention for pulmonary fibrosis via mitochondrial transfer promoted by mitochondrial biogenesis
Source: Nat Commun. 2023 Sep 18;14:5781. doi: 10.1038/s41467-023-41529-7 (PMC10507082; doi:10.1038/s41467-023-41529-7)
Supplement: Supplementary file 1 — Supplementary Information [file 41467_2023_41529_MOESM1_ESM.pdf]

Efficient intervention for pulmonary fibrosis via mitochondrial transfer promoted by mitochondrial biogenesis

Table of Contents

Supplementary Fig. S1. Optimisation of using the treatment with Pg to increase the mitochondrial mass in hMSC.
Supplementary Fig. S2. Impacts of the treatment with Pg on mitochondrial transfer rate.
Supplementary Fig. S3. Both the expression levels of PGC-1*α* and Cx43 determine the efficiency of mitochondrial transfer rate.
Supplementary Fig. S4. TEM images of incorporated mitochondria in an annular gap junction.
Supplementary Fig. S5. Impacts of the engineering with Pg and IONPs on multiple differentiation potentials of hMSC.
Supplementary Fig. S6. Dynamic observations of the mitochondrial transfer process with hMSC.
Supplementary Fig. S7. Dynamic observations of the mitochondrial transfer process with Pg-Fe-hMSC.
Supplementary Fig. S8. Enlarged images of the above dynamic transfer process of mitochondria 2.5 h after the co-incubation.
Supplementary Fig. S9. Intercellular mitochondrial transfer from hMSCs to different receiving cells.
Supplementary Fig. S10. Limited mitochondrial transfer rate between healthy cells.
Supplementary Fig. S11. Mitochondrial transfer rates of different engineered hMSCs.
Supplementary Fig. S12. Gating strategy by flow cytometers for quantitative determinations of mitochondrial transfer rates.
Supplementary Fig. 13. Observations of long-term mitochondrial transfer from different engineered hMSCs to BLM-TC-1 cells.
Supplementary Fig. 14. Direct treatment of TC-1 cells with Pg has poor protection against the injury caused by BLM.
Supplementary Fig. S15. Cell number ratios impact the mitochondrial transfer rates and associated therapeutic effects.
Supplementary Fig. S16. The severity of injury of receiving cell affects the intercellular mitochondrial transfer capacity.

Supplementary Fig. S17. Pg-Fe-hMSC demonstrates a more powerful therapeutic effect on TC-1 cell with more severe injury.
Supplementary Fig. S18. Morphological observation of mitochondria in mice lung cells after the indicated treatments using TEM.
Supplementary Fig. S19. H&E staining of major organs in C57BL/6 mice with and without Pg-Fe-hMSC treatment.
Supplementary Fig. S20. Preliminary safety evaluations on the treatment using Pg-Fe-hMSC.
Supplementary Fig. S21. Cell viability of primary human lung epithelial cells after the treatment with BLM at different concentrations.
Supplementary Fig. S22. Cell viability and TGF-β expression levels of 3D multicellular humanised lung model post the treatment with BLM at different concentrations.
Supplementary Fig. S23. Confirmation of the fibrotic 3D multicellular humanised lung model.
Supplementary Fig. S24. Distribution of Pg-Fe-hMSC in the fibrotic 3D multicellular humanised lung spheroid.
Supplementary Fig. S25. Observation of the co-localisation of mitochondria and lysosomes.
Supplementary Fig. S26. Pg-Fe-hMSC promotes the autophagy of BLM-TC-1 cell.
Supplementary Table S1. STR authentication of human umbilical vein endothelial cells (HUVEC)
Supplementary Table S2. STR authentication of BEAS-2B cells
Supplementary Table S3. STR authentication of Hs888Lu cells
Supplementary Table S4. Primer sequences for PCR
Supplementary Table S5. Antibody information

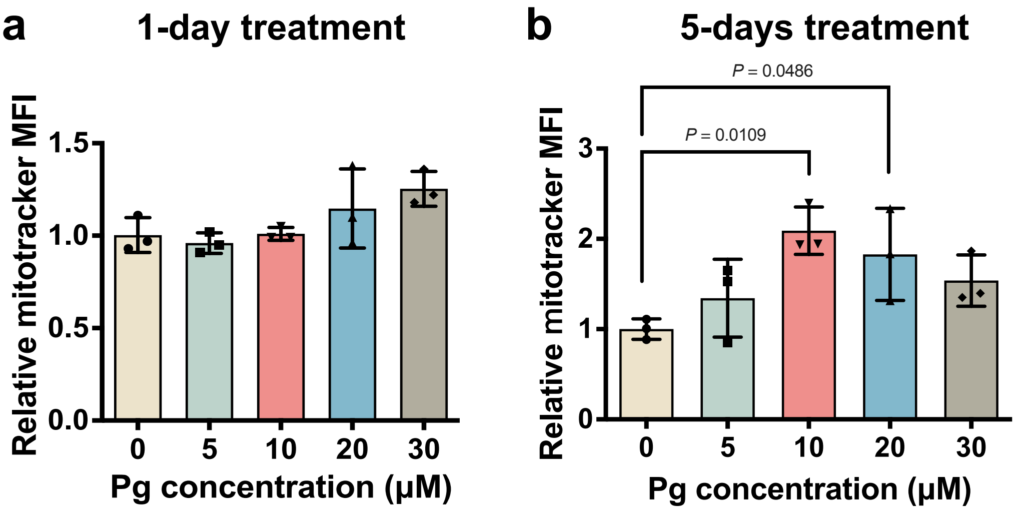

Supplementary Fig. S1. Optimisation of using the treatment with Pg to increase the mitochondrial mass in hMSC. (a) Mitochondrial mass indicated by the mean fluorescence index (MFI) of the mitochondrial tracker in hMSC after the treatment with Pg at different concentrations for 1 day (n = 3 biologically independent cells). (b) Mitochondrial mass in hMSC after the treatment with Pg at different concentrations for five consecutive days (n = 3 biologically independent cells). Data are presented as means ± SD. Statistical significance was analysed using ordinary one-way analysis of variance (ANOVA).

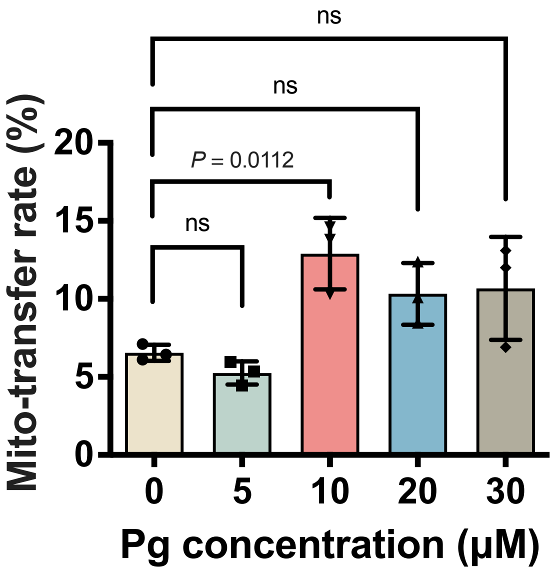

Supplementary Fig. S2. Impacts of the treatment with Pg on mitochondrial transfer rate. The mitochondrial transfer rate of hMSC after the treatment with Pg at different concentrations for 5 consecutive days (n = 3 biologically independent cells). Data are presented as means ± SD. Statistical significance was analysed using ordinary one-way ANOVA. ns, no significant difference.

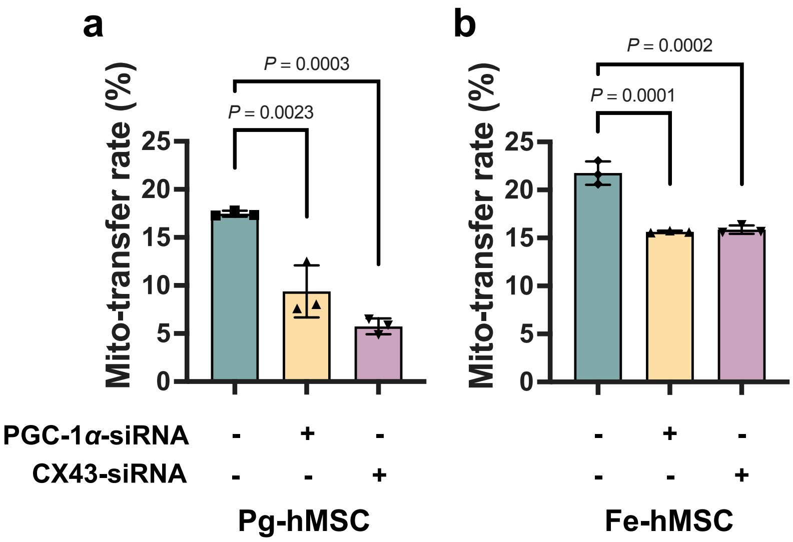

Supplementary Fig. S3. Both the expression levels of PGC-1*α* and Cx43 determine the efficiency of mitochondrial transfer rate. (a) The mitochondrial transfer rate of Pg-hMSC with indicated treatments (n = 3 biologically independent cells). (b) The mitochondrial transfer rate of Fe-hMSC with indicated treatments (n = 3 biologically independent cells). Data are presented as means ± SD. Statistical significance was analysed using ordinary one-way ANOVA.

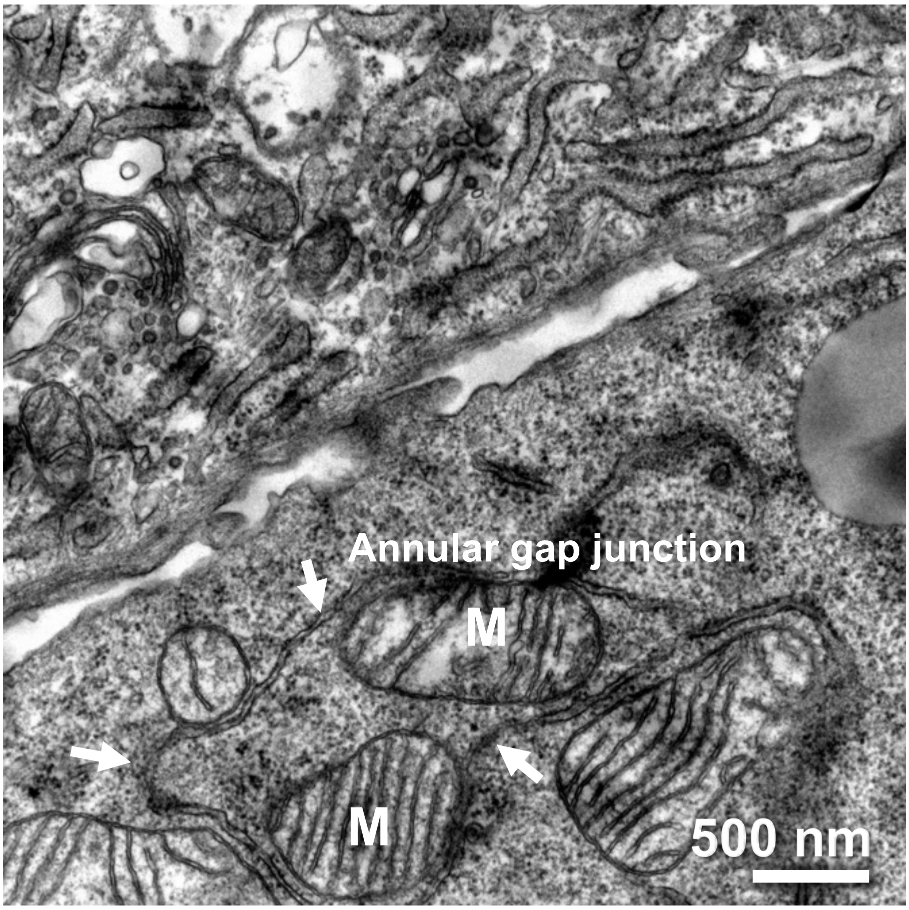

Supplementary Fig. S4. TEM images of incorporated mitochondria in an annular gap junction. A typical double-membrane annular gap junction structure with incorporated mitochondria is observed after the mitochondrial transfer. Scale bar, 500 nm.

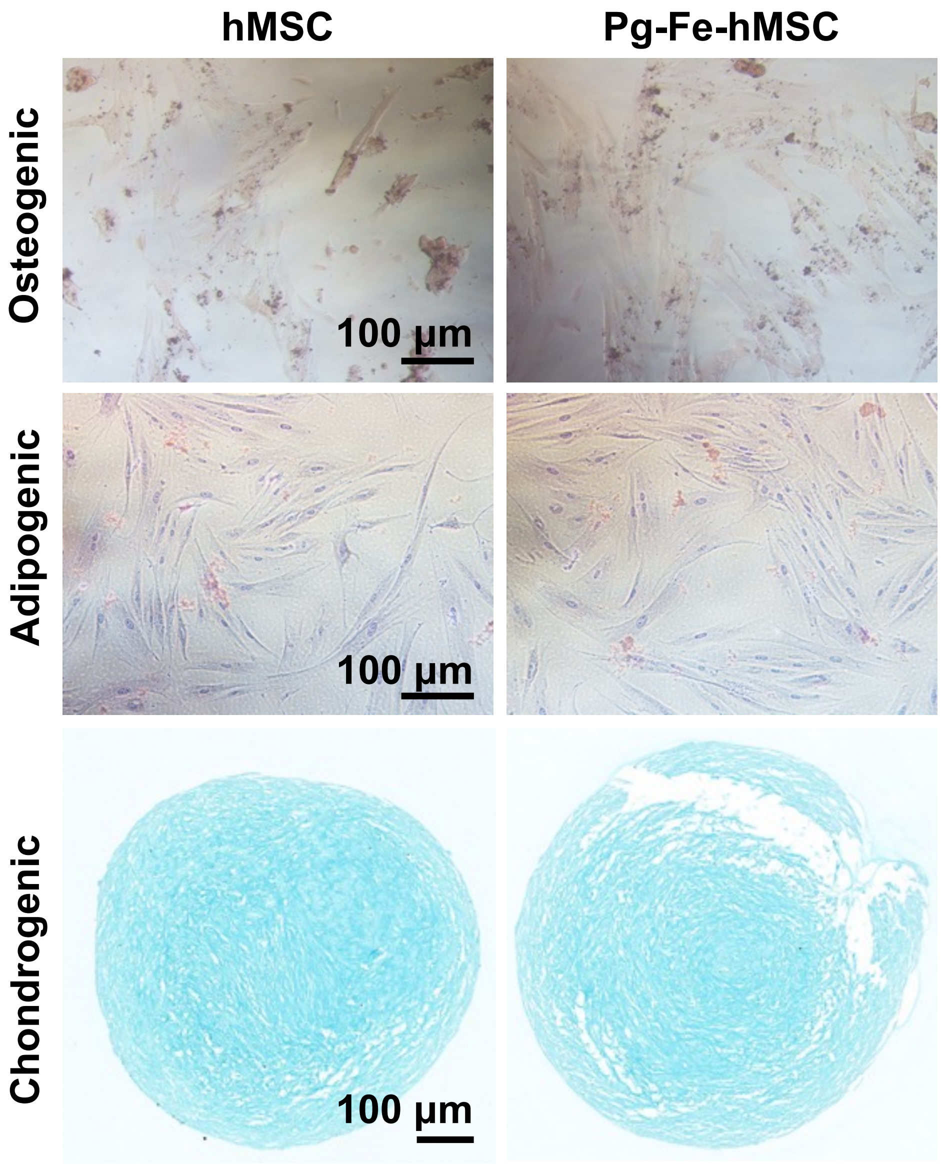

Supplementary Fig. S5. Impacts of the engineering with Pg and IONPs on multiple differentiation potentials of hMSC. Differentiations of Pg-Fe-hMSC to osteocytes, adipocytes and chondrocytes were not impacted. Scale bars, 100 µm.

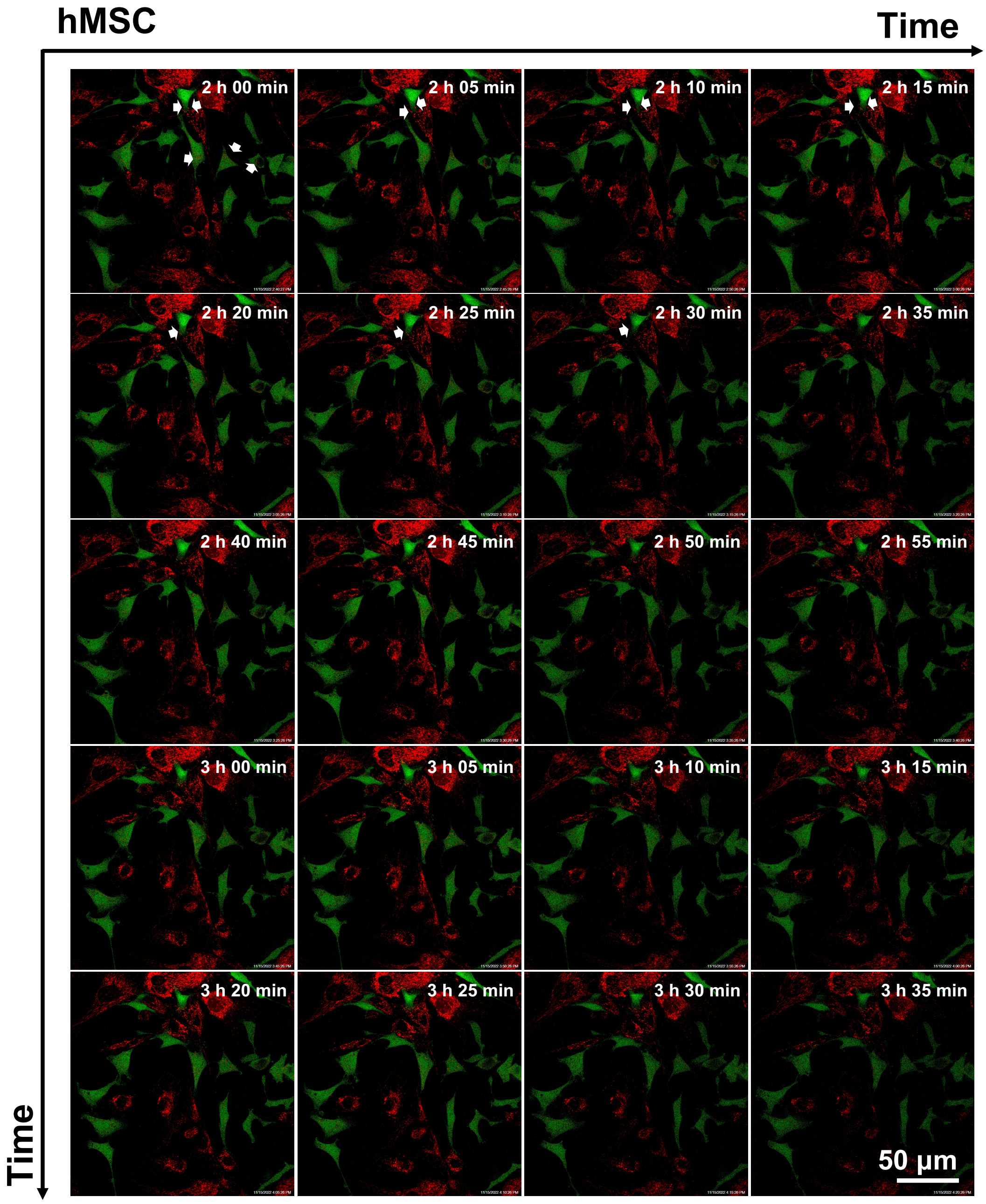

Supplementary Fig. S6. Dynamic observations of the mitochondrial transfer process with hMSC. Limited number of mitochondria was observed to be transferred from hMSC to BLM-TC-1 cells. Green: BLM-TC-1; red: mitochondria of hMSC; white arrows indicate the transferred mitochondria. Scale bar, 50 μm.

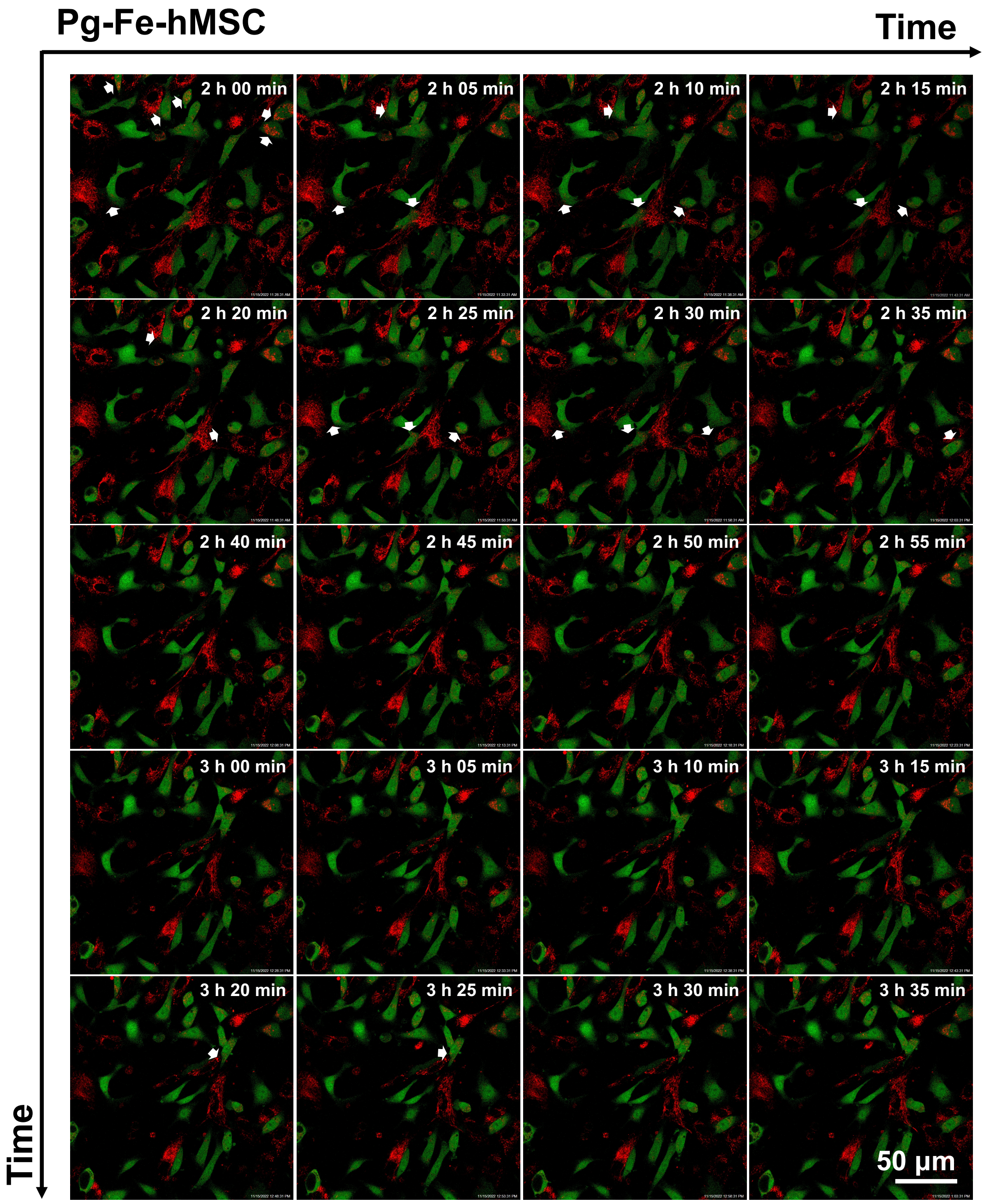

Supplementary Fig. S7. Dynamic observations of the mitochondrial transfer process with Pg-Fe-hMSC. Increased number of mitochondria was observed to be transferred from Pg-Fe-hMSC to BLM-TC-1 cells within a prolonged time period. Green: BLM-TC-1; red: mitochondria of Pg-Fe-hMSC; white arrows indicate the transferred mitochondria. Scale bar, 50 μm.

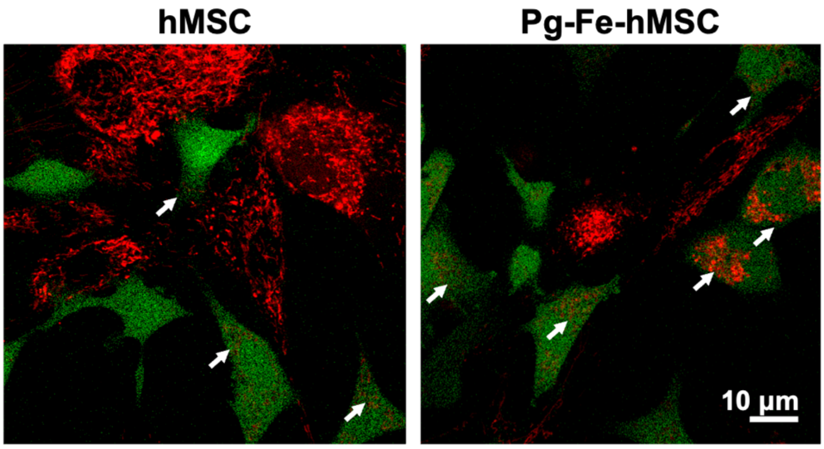

Supplementary Fig. S8. Enlarged images of the above dynamic transfer process of mitochondria 2.5 h after the co-incubation. Transferred mitochondria from hMSC or Pg-Fe-hMSC to BLM-TC-1 cells were indicated by white arrows. Pg-Fe-hMSC had a higher mitochondrial transfer rate than hMSC. Green: BLM-TC-1; red: mitochondria of hMSC; white arrows indicate the transferred mitochondria. Scale bar, 10 μm.

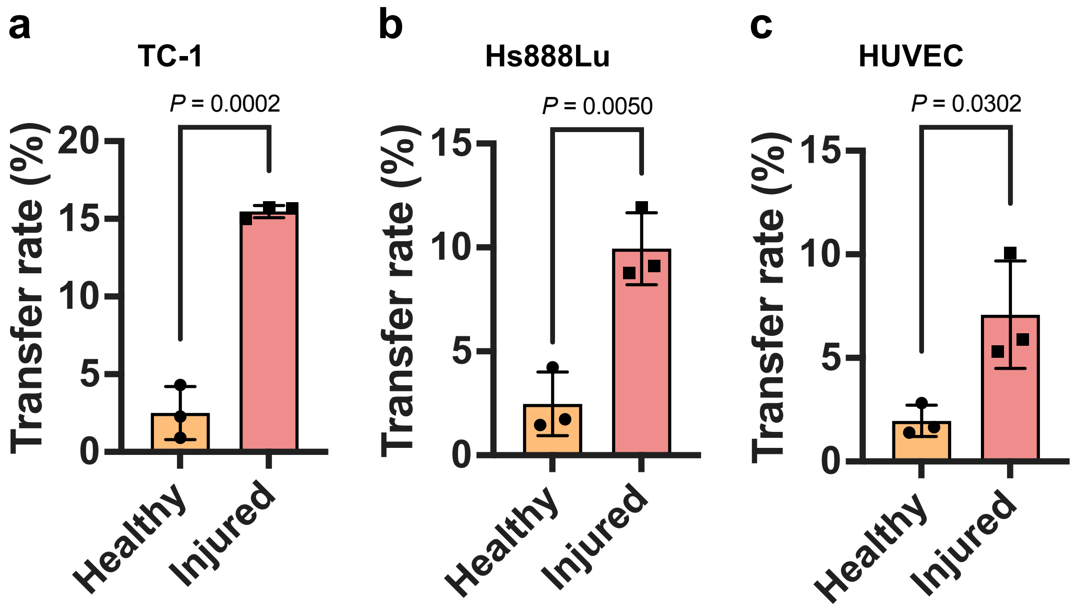

Supplementary Fig. S9. Intercellular mitochondrial transfer from hMSCs to different receiving cells. (a) Mitochondrial transfer rate from hMSCs to healthy and injured TC-1 cells (n = 3 biologically independent cells). (b) Mitochondrial transfer rate from hMSCs to healthy and injured Hs888Lu cells (n = 3 biologically independent cells). (c) Mitochondrial transfer rate from hMSCs to healthy and injured human umbilical vein endothelial cells (HUVECs) (n = 3 biologically independent cells). Data are presented as means ± SD. Statistical significance was analysed using two-sided Student's t-test.

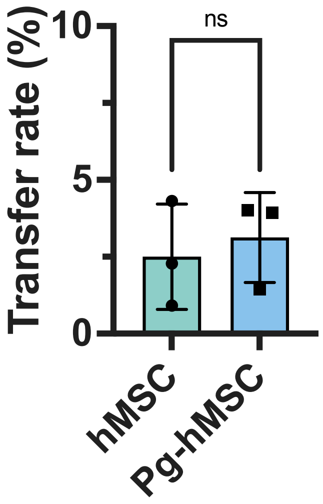

Supplementary Fig. S10. Limited mitochondrial transfer rate between healthy cells. Mitochondrial transfer rate from hMSC or Pg-hMSC towards healthy TC-1 cell (n = 3 biologically independent cells). Data are presented as means ± SD. Statistical significance was analysed using two-sided Student's t-test. ns, no significant difference.

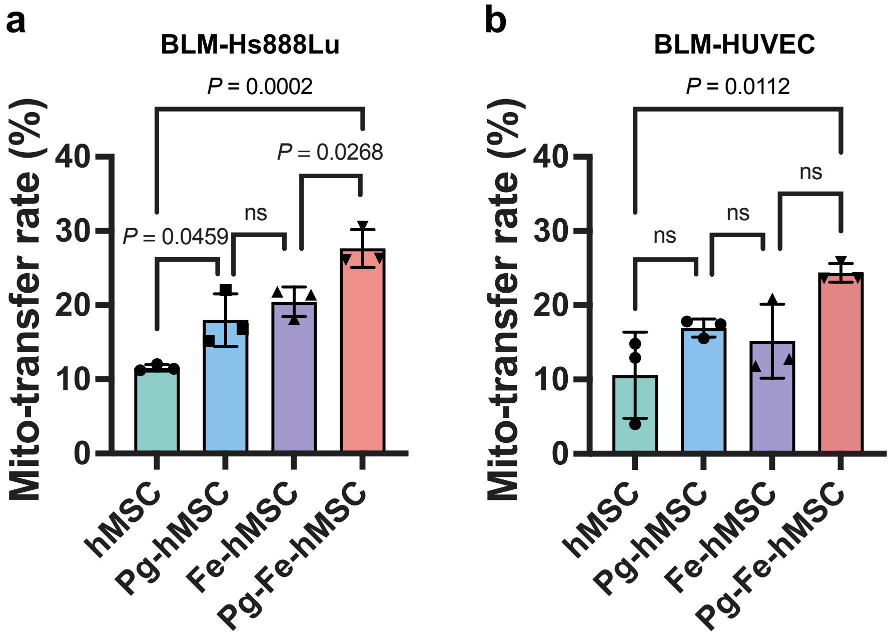

Supplementary Fig. S11. Mitochondrial transfer rates of different engineered hMSCs. (a) Mitochondrial transfer rates of different engineered hMSCs to BLM-treated Hs888Lu cells (n = 3 biologically independent cells). (b) Mitochondrial transfer rates of different engineered hMSCs to BLM-treated HUVECs (n = 3 biologically independent cells). Data are presented as means ± SD. Statistical significance was analysed using ordinary one-way ANOVA. ns, no significant difference.

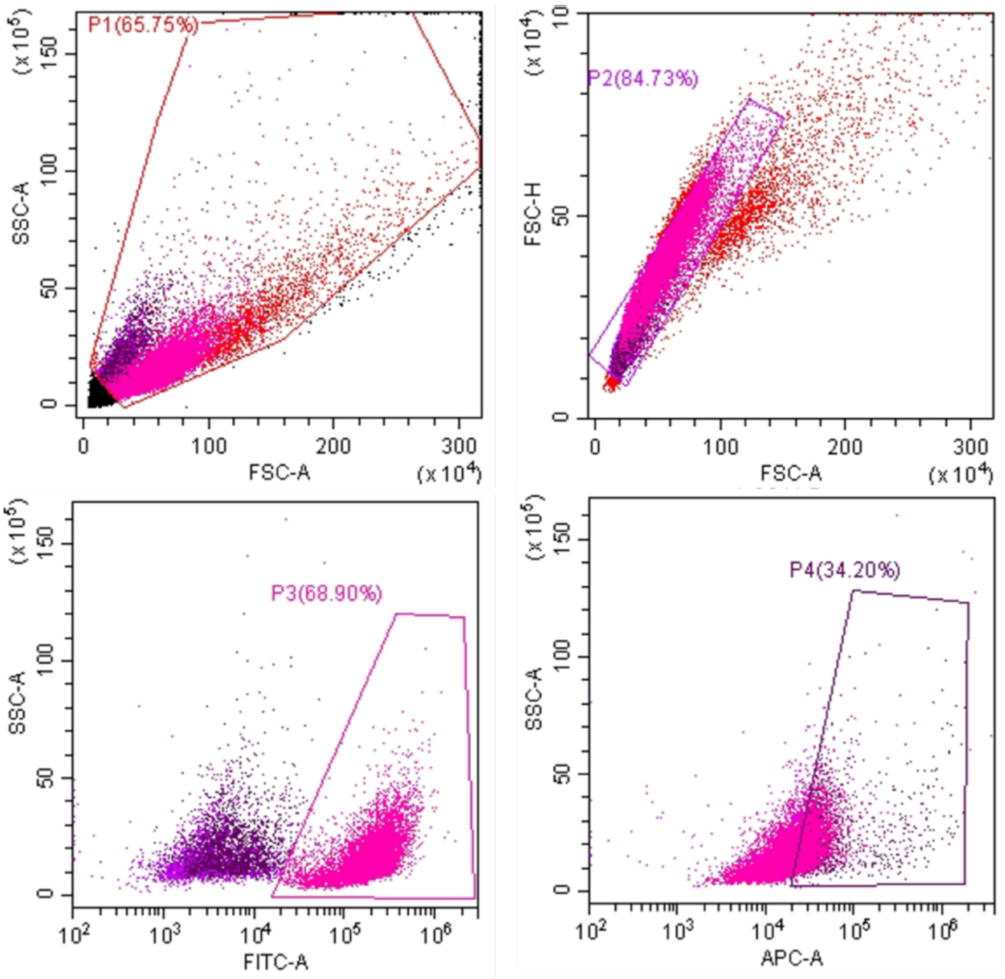

Supplementary Fig. S12. Gating strategy by flow cytometers for quantitative determinations of mitochondrial transfer rates.

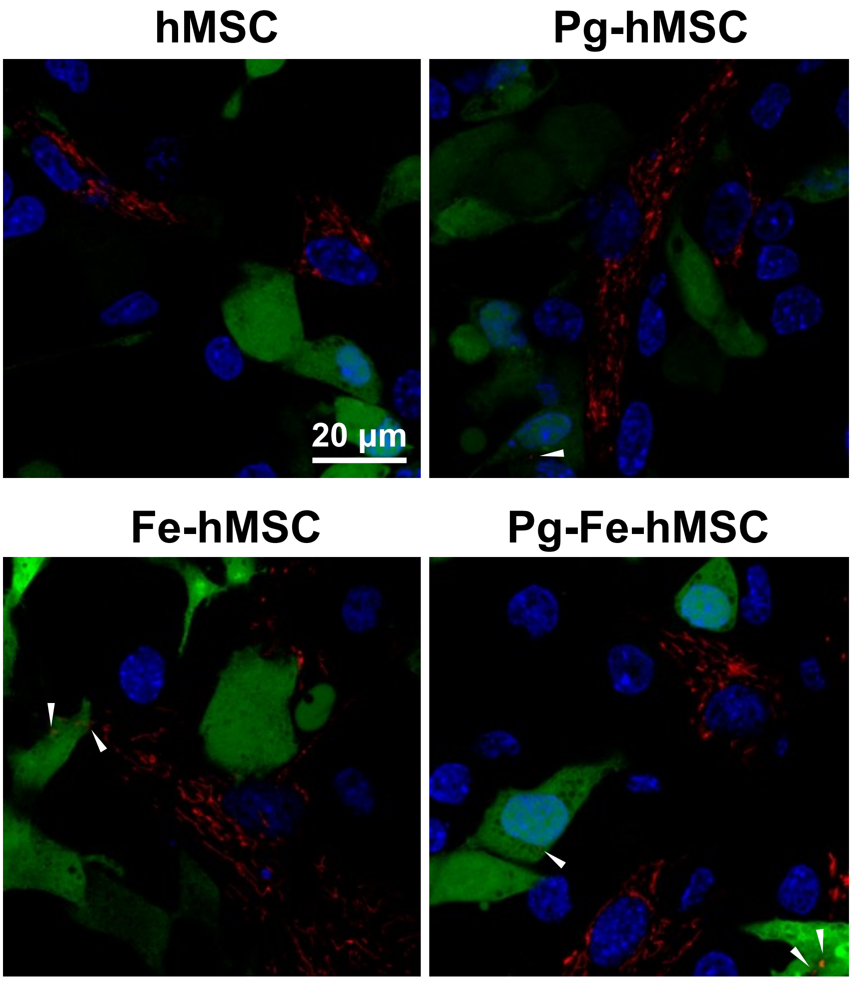

Supplementary Fig. 13. Observations of long-term mitochondrial transfer from different engineered hMSCs to BLM-TC-1 cells. Mitochondrial transfer at 96 h post the co-culture by using fluorescence microscopy. Blue: nuclei; green: BLM-TC-1; red: mitochondria of hMSC; white arrows indicate the transferred mitochondria. Scale bar, 20 µm.

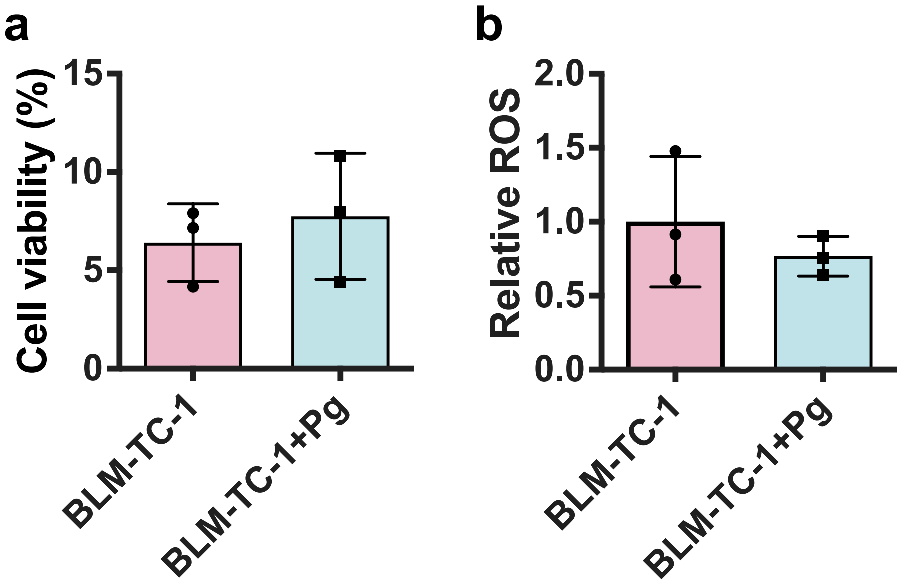

Supplementary Fig. 14. Direct treatment of TC-1 cells with Pg has poor protection against the injury caused by BLM. (a) Cell viability of BLM-TC-1 cells with and without Pg treatment (n = 3 biologically independent cells). (b) Relative ROS levels of BLM-TC-1 cells with and without Pg treatments (n = 3 biologically independent cells). Data are presented as means ± SD. Statistical significance was analysed using two-sided Student's t-test.

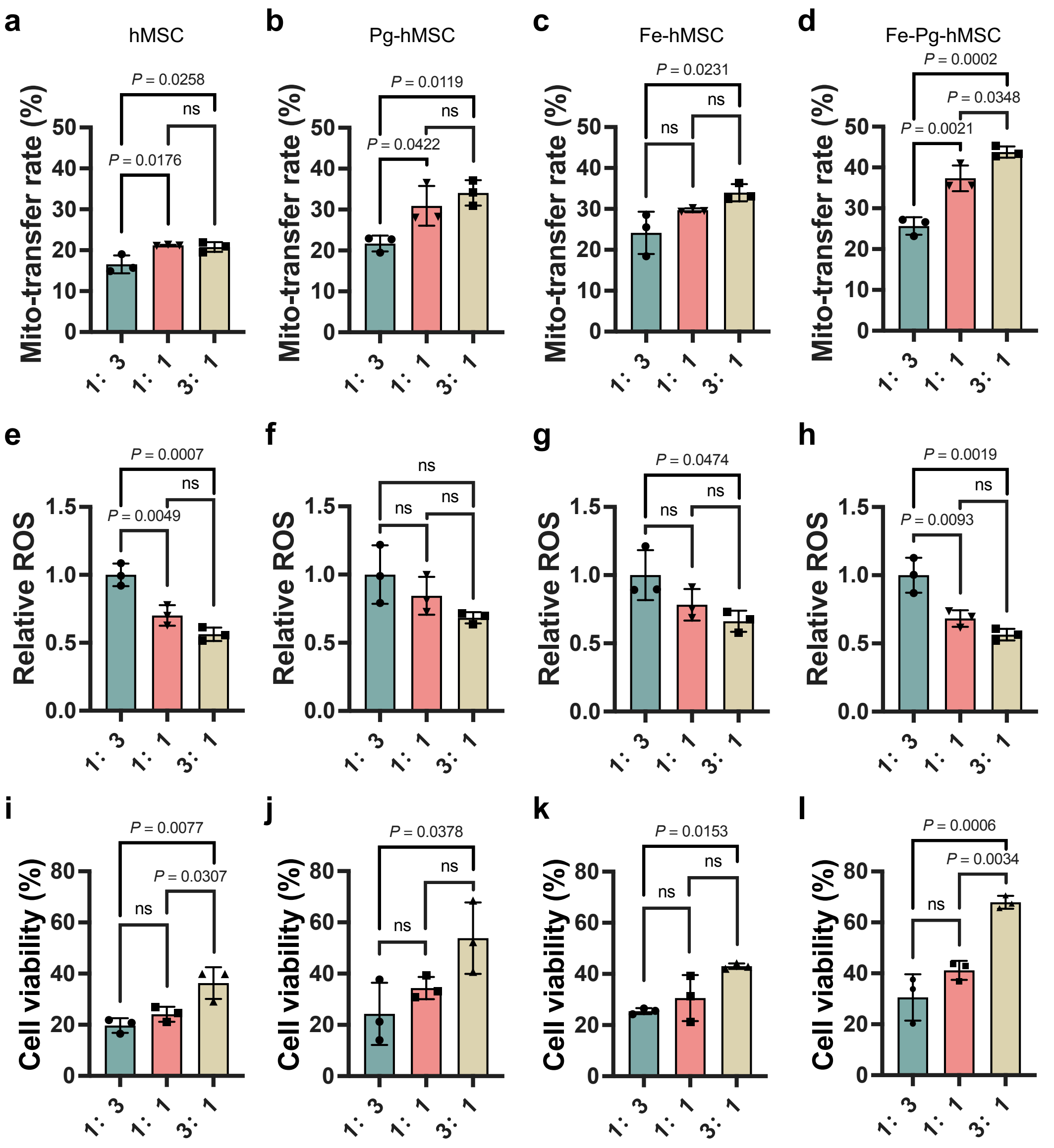

Supplementary Fig. S15. Cell number ratios impact the mitochondrial transfer rates and associated therapeutic effects. Mitochondrial transfer rates of (a) hMSC, (b) Pg-hMSC, (c) Fe-hMSC, and (d) Pg-Fe-hMSC at the indicated cell number ratios for co-culture (different engineered hMSCs:BLM-TC-1) (n = 3 biologically independent cells for each). Relative ROS levels of BLM-TC-1 after co-culture with (e) hMSC, (f) Pg-hMSC, (g) Fe-hMSC, and (h) Pg-Fe-hMSC at the indicated cell number ratios (n = 3 biologically independent cells for each). Cell viability of BLM-TC-1 after co-culture with (i) hMSC, (j) Pg-hMSC, (k) Fe-hMSC, and (l) Pg-Fe-hMSC at the indicated cell number ratios (n = 3 biologically independent cells for each). Data are presented as means ± SD. Statistical significance was analysed using ordinary one-way ANOVA.

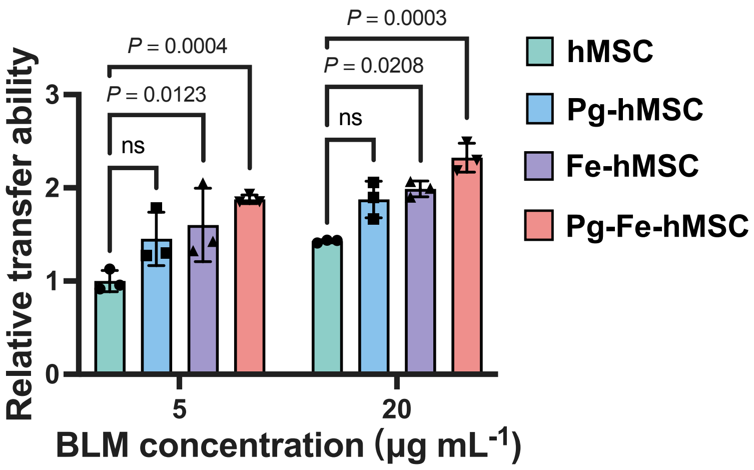

Supplementary Fig. S16. The severity of injury of receiving cell affects the intercellular mitochondrial transfer capacity. Comparisons of mitochondrial transfer capacity from different engineered hMSCs to injured TC-1 cells induced by the treatment of BLM at indicated concentrations. The mitochondrial transfer rate of hMSC was regarded as the base-line and was compared with the transfer rate of Pg-hMSC, Fe-hMSC, and Pg-Fe-hMSC (n = 3 biologically independent cells). Data are presented as means ± SD. Statistical significance was analysed using ordinary one-way ANOVA. ns, no significant difference.

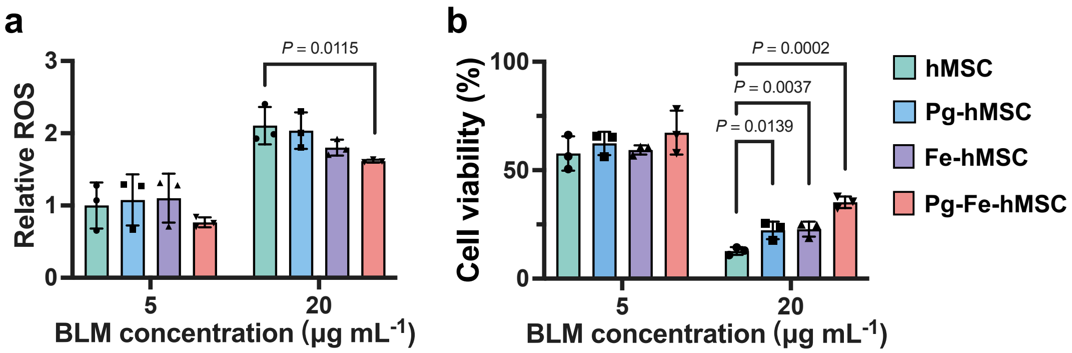

Supplementary Fig. S17. Pg-Fe-hMSC demonstrates a more powerful therapeutic effect on TC-1 cell with more severe injury. (a) Relative ROS level and (b) Cell viability of BLM-TC-1 cells post the treatments with different engineered hMSCs (n = 3 biologically independent cells for each). Data are presented as means ± SD. Statistical significance was analysed using ordinary one-way ANOVA.

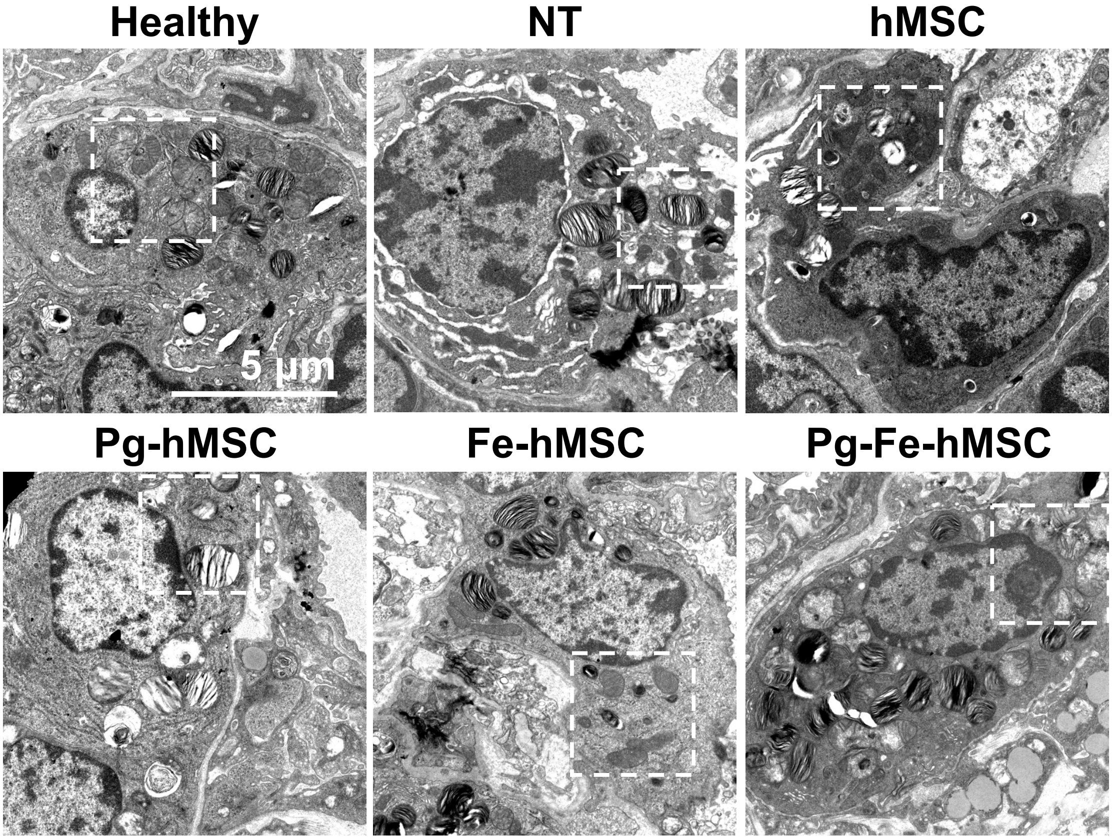

Supplementary Fig. S18. Morphological observation of mitochondria in mice lung cells after the indicated treatments using TEM. TEM images of Fig. 4g in a larger scale. White dotted squares indicate the enlarged area in Fig. 4g. Scale bar, 5 μm.

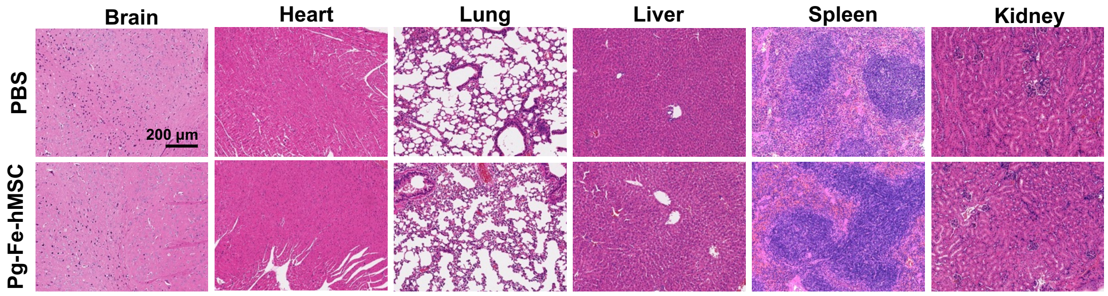

Supplementary Fig. S19. H&E staining of major organs in C57BL/6 mice with and without Pg-Fe-hMSC treatment. No obvious pathological changes were observed post Pg-Fe-hMSC treatment. Scale bar, 200 μm.

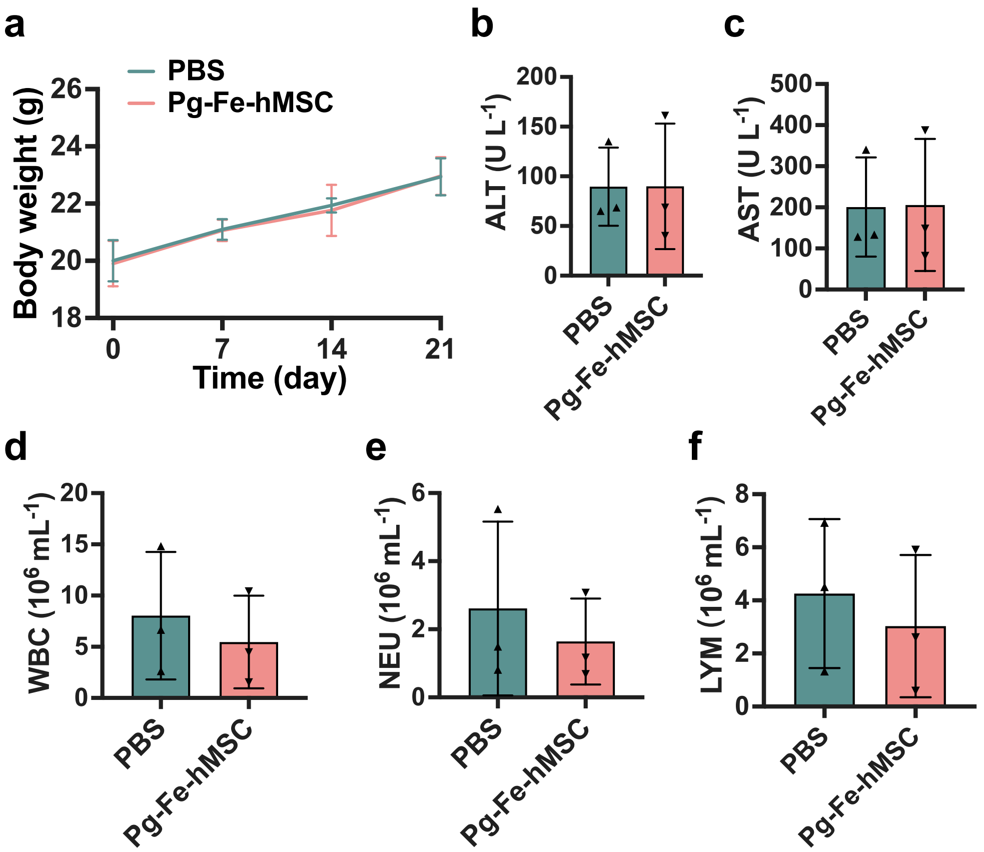

Supplementary Fig. S20. Preliminary safety evaluations on the treatment using Pg-Fe-hMSC. (a) Body weight changes of C57BL/6 mice with and without Pg-Fe-hMSC treatment (n = 3 biologically independent animals). (b) Alanine transaminase (ALT), (c) Aspartate transaminase (AST), (d) White blood cell (WBC), (e) Neutrophil (NEU), and (f) Lymphocyte (LYM) counts of mice with and without Pg-Fe-hMSC treatments (n = 3 biologically independent animals for each). Data are presented as means ± SD. Statistical significance was analysed using Student's t-test.

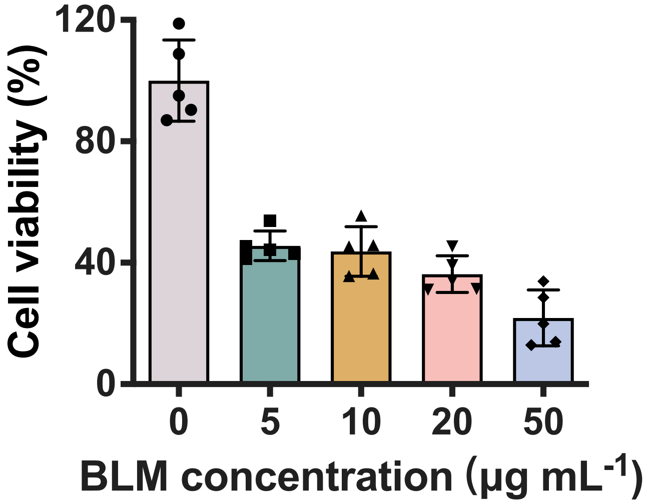

Supplementary Fig. S21. Cell viability of primary human lung epithelial cells after the treatment with BLM at different concentrations. Cell viability was evaluated 24 h after the treatment with BLM (n = 5 biologically independent cells). Data are presented as means ± SD.

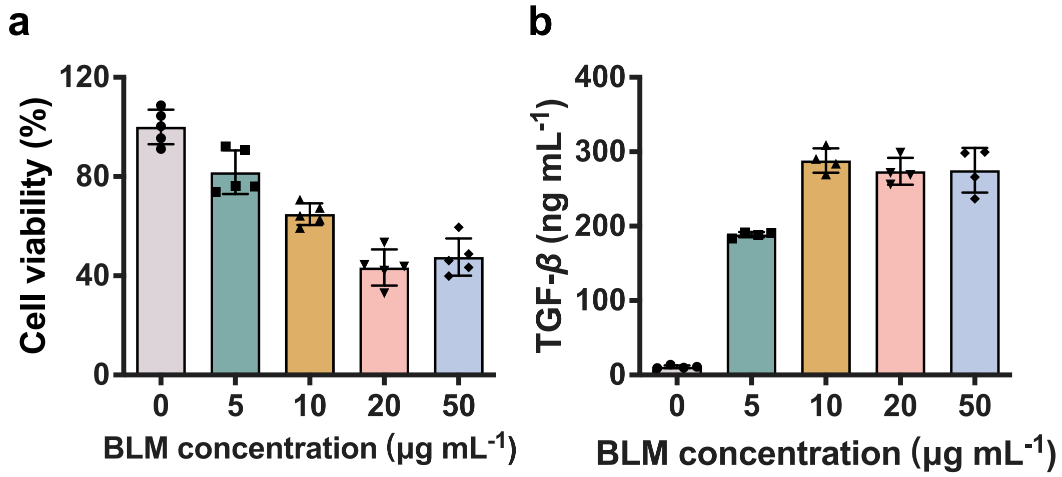

Supplementary Fig. S22. Cell viability and TGF-*β* expression levels of 3D multicellular humanised lung model post the treatment with BLM at different concentrations. (a) Cell viability of 3D humanised lung model after the treatment with BLM at the indicated concentrations (n = 5 biologically independent samples). (b) TGF-*β* levels of 3D humanised lung model after the treatment with BLM at the indicated concentrations (n = 4 biologically independent samples). Data are presented as means ± SD.

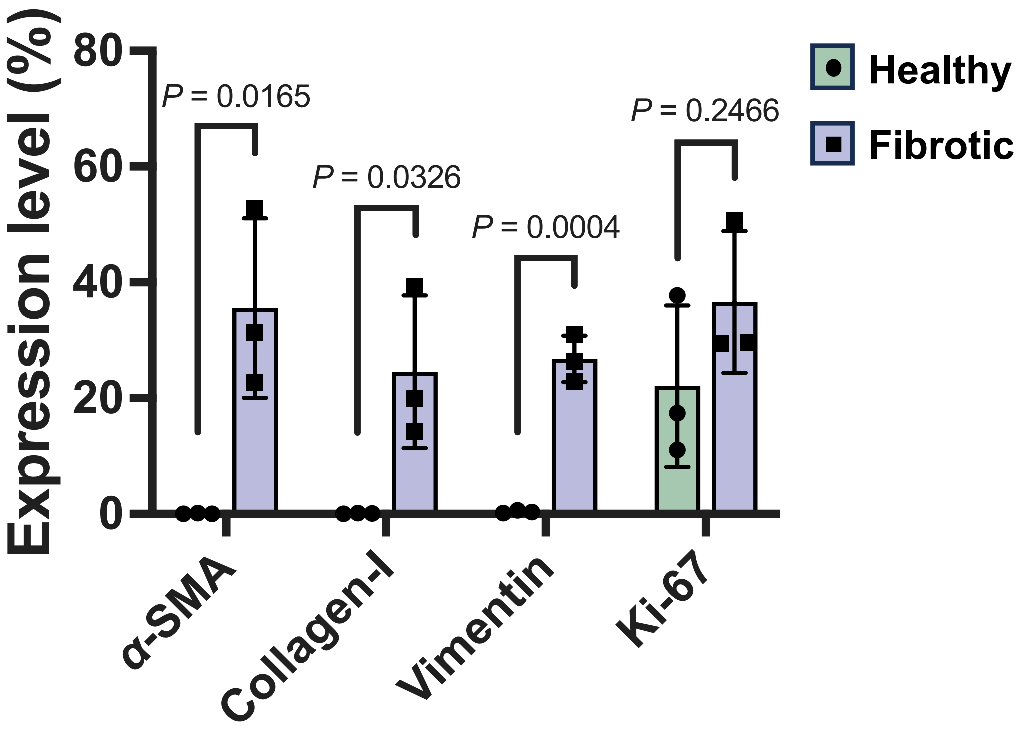

Supplementary Fig. S23. Confirmation of the fibrotic 3D multicellular humanised lung model. The expression levels of *α*-SMA, vimentin, collagen-I, and Ki-67 after immunofluorescent staining were calculated according to the density of fluorescence signals using Image J software (n = 3 biologically independent samples). Data are presented as means ± SD. Statistical significance was analysed using ordinary one-way ANOVA.

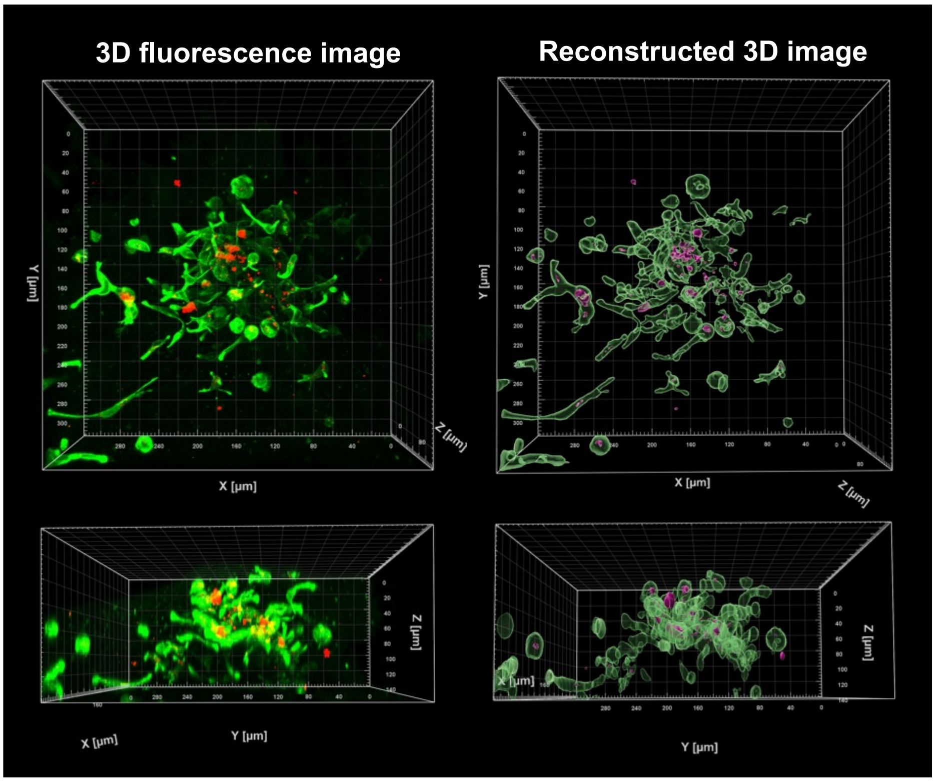

Supplementary Fig. S24. Distribution of Pg-Fe-hMSC in the fibrotic 3D multicellular humanised lung spheroid. Fluorescent signals of Pg-Fe-hMSC were observed inside the 3D fibrotic lung spheroid (left panel). The distribution images were then reconstructed according to the fluorescent signals and were demonstrated in the right panel. Green: human bronchial epithelial cells and lung fibroblasts; red: mitochondria of Pg-Fe-hMSC.

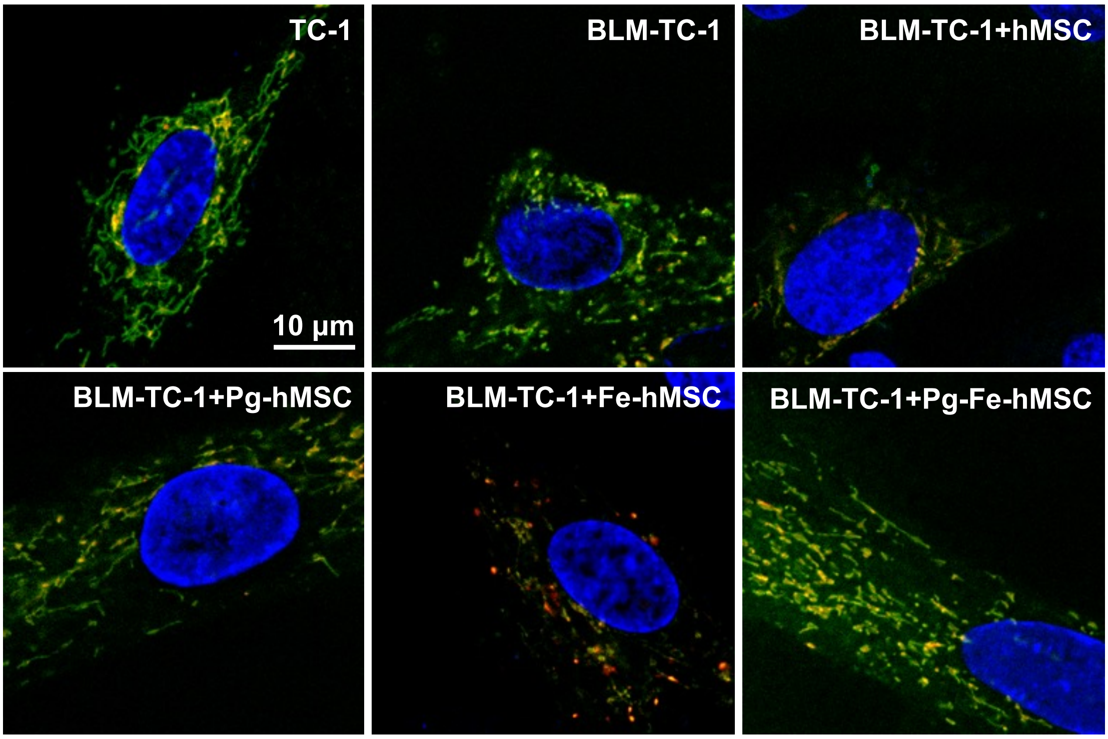

Supplementary Fig. S25. Observation of the co-localisation of mitochondria and lysosomes. Inhibited co-localisation of mitochondria and lysosomes in BLM-TC-1 cells was relieved after receiving exogenous mitochondria transferred from different engineered hMSCs, especially from Pg-Fe-hMSC. Blue: cell nuclei; green: mitochondria; red: lysosomes. Scale bar, 10 μm.

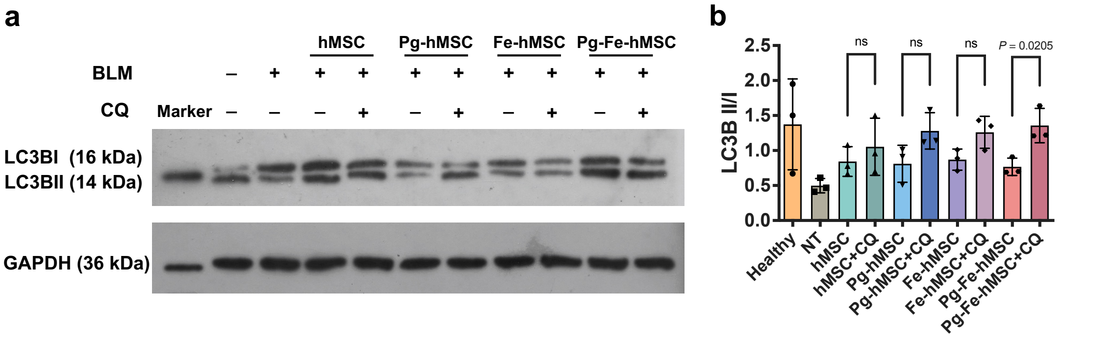

Supplementary Fig. S26. Pg-Fe-hMSC promotes the autophagy of BLM-TC-1 cell. (a) Representative Western blot images of LC3B expression level by TC-1 cells with or without chloroquine (CQ) treatments. (b) Semiquantitative determination of the Western blot analysis (n = 3 biologically independent samples). Data are presented as means ± SD. Statistical significance was analysed using ordinary one-way ANOVA. ns, no significant difference.

Supplementary Table S1. STR authentication of human umbilical vein endothelial cells (HUVEC)

Maker	Sample information				Cell Bank information	
	Allele1	Allele2	Allele3	Allele4	Allele1	Allele2
D5S818	11	12			11	12
D13S317	9	11			9	11
D7S820	8	12			8	12
D16S539	11	12			11	12
VWA	16	16			16	16
TH01	6	9.3			6	9.3
AMEL	X	X			X	X
TPOX	8	11			8	11
CSF1PO	11	12			11	12
D12S391	17	17				
FGA	21	23				
D2S1338	18	22				
D21S11	28	31				
D18S51	13	17				
D8S1179	15	17				
D3S1358	16	16				
D6S1043	12	18				
PENTAE	7	13				
D19S433	12	13				
PENTAD	12	13				
D1S1656	12	17.3				

Supplementary Table S2. STR authentication of BEAS-2B cells

Maker	Sample information				Cell Bank information	
	**Allele1**	**Allele2**	**Allele3**	**Allele4**	**Allele1**	**Allele2**
D5S818	12	13			12	13
D13S317	13	13			13	13
D7S820	10	13			10	13
D16S539	12	12			12	12
VWA	17	18			17	18
TH01	7	9.3			7	9.3
AMEL	X	Y			X	Y
TPOX	6	11			6	11
CSF1PO	9	12			9	12
D12S391	17	18				
FGA	20	24				
D2S1338	22	23				
D21S11	28	30				
D18S51	18	19				
D8S1179	13	15				
D3S1358	15	17				
D6S1043	12	18				
PENTAE	5	8				
D19S433	13.2	15.2				
PENTAD	3	13				
D1S1656	14	15				

Supplementary Table S3. STR authentication of Hs888Lu cells

Maker	Sample information				Cell Bank information	
	Allele1	Allele2	Allele3	Allele4	Allele1	Allele2
D5S818	12	13			13	13
D13S317	11	14			11	14
D7S820	7	11			7	11
D16S539	8	12			8	12
VWA					15	18
TH01	9.3	9.3			10	9.3
AMEL	X	Y			X	Y
TPOX	8	11			8	11
CSF1PO	10	11			10	11
D12S391	19	20				
FGA	21	23				
D2S1338	17	18	19	20		
D21S11	28	29				
D18S51	13	16				
D8S1179						
D3S1358	16	17	18			
D6S1043	12	19				
PENTAE	13	19				
D19S433	13	14				
PENTAD	13	13				

Supplementary Table S4. Primer sequences for PCR

Gene	Genbank Accession	Primer Sequences (5' to 3')
Human GAPDH	NM_002046.5	CCATGACAACTTTGGTATCGTGGAA<br>GGCCATCACGCCACAGTTTC
Mouse GAPDH	GU214026.1	GAAGGTCGGTGTGAACGGATTTG<br>CATGTAGACCATGTAGTTGAGGTCA
Human TFAM	NM_003201.3	GCTCCCCCTTCAGTTTTGTG<br>CTCCAACGCTGGGCAATTC
Human PGC-1α	NM_001330751.2	GTCCTCACAGAGACACTAGACA<br>CTCCATCTGTCAGCGCATCAA
Mouse Collagen-I	NM_007743.2	CCTTCTGGACCCGTTGGCAAAGAT<br>GGCTACCCTGAGAACCACGAACA
Human NRF1	NM_005011.5	TGTATCTCACCCTCCAAACCTAACCG<br>CACCACATTCTCCAAAGG
Mouse Tomm5	NM_001099675.1	GGAGGAGATGAAGCGGAAGAT<br>AGGGCCACGTAGATGAGGAA
Mouse Tomm6	NM_001164729.1	GCCTCAGAGGTTCCAGACAA<br>CCTCCGGAAGTCGTTTCGAT
Mouse Tomm70	NM_138599.5	GAGCAGAGCATGCTGTTAGCT<br>GCATCAGAGGTTCACGGTTCT
Mouse Tomm5	NM_001099675.1	GGAGGAGATGAAGCGGAAGAT<br>AGGGCCACGTAGATGAGGAA
Mouse Tomm6	NM_001164729.1	GCCTCAGAGGTTCCAGACAA<br>CCTCCGGAAGTCGTTTCGAT
Mouse ND1	NC_005089.1:2751-3707	CAGCCTGACCCATAGCCATA<br>CCGGCTGCGTATTCTACGTT
Mouse ND3	NC_005089.1:9459-9806	GCGGATTCGACCCTACAAG<br>GCTCATGGTAGTGGAAGTAGAAGA
Mouse Cox1	NC_005089.1:5328-6872	CCCCAGCCATAACACAGTATC<br>CACTGGTAGTGATAATAGGAGCAGT
Mouse Cox2	NM_011198.4	GGCAGGAAGTCTTTGGTCTGGT<br>CTGGTTTGGAATAGTTGCTCATCAC

Supplementary Table S5. Antibody information

Antibody	Source	Cat. no.	Dilution
Mouse anti-CD31 FITC	Biolegend	102405	1:200
Mouse anti-CD45 PerCP/Cy5.5	Biolegend	103132	1:200
Mouse anti-CD326 PE	Biolegend	118205	1:200
Goat anti-rabbit IgG (H+L) Secondary Antibody FITC	Boster	BA1105	1:100
Rabbit anti-EpCAM	Affinity	DF6311	1:500
Rabbit anti-Ki67	Boster	PB9026	1:200
Rabbit anti-VIM	Boster	BM4029	1:100
Rabbit anti-*α*-SMA	Boster	BM3902	1:100
Rabbit anti-collagen, type 1	Proteintech	14695-1-AP	1:500
Rabbit anti-LC3B	CST	43566	1:1000
Rabbit anti-GAPDH	Abcam	Ab181602	1:10000
Goat anti-rabbit IgG (H+L)	Thermo Pierce	31210	1:5000
Goat anti-mouse IgG (H+L)	Thermo Pierce	31431	1:5000
