# Supplementary material for: Efficient intervention for pulmonary fibrosis via mitochondrial transfer promoted by mitochondrial biogenesis
Source: Nat Commun. 2023 Sep 18;14:5781. doi: 10.1038/s41467-023-41529-7 (PMC10507082; doi:10.1038/s41467-023-41529-7)
Supplement: Supplementary file 3 — Description of additional supplementary files [file 41467_2023_41529_MOESM3_ESM.pdf]

Description of Additional Supplementary Files Document

Supplementary Movie S1.

Dynamic observations of the mitochondrial transfer process from hMSC to BLM-TC-1 cells.

Supplementary Movie S2.

Dynamic observations of the mitochondrial transfer process from Pg-Fe-hMSC to BLM-TC1 cells.
