# Supplementary material for: Efficient intervention for pulmonary fibrosis via mitochondrial transfer promoted by mitochondrial biogenesis
Source: Nat Commun. 2023 Sep 18;14:5781. doi: 10.1038/s41467-023-41529-7 (PMC10507082; doi:10.1038/s41467-023-41529-7)
Supplement: Supplementary file 6 — Reporting Summary [file 41467_2023_41529_MOESM6_ESM.pdf]

Corresponding author(s): Jianqing Gao, Tianyuan Zhang

Last updated by author(s): Aug 15, 2023

Reporting Summary

Nature Portfolio wishes to improve the reproducibility of the work that we publish. This form provides structure for consistency and transparency in reporting. For further information on Nature Portfolio policies, see our Editorial Policies and the Editorial Policy Checklist.

Statistics

For all statistical analyses, confirm that the following items are present in the figure legend, table legend, main text, or Methods section.

n/a	Confirmed	
☐	☒	The exact sample size (*n*) for each experimental group/condition, given as a discrete number and unit of measurement
☐	☒	A statement on whether measurements were taken from distinct samples or whether the same sample was measured repeatedly
☐	☒	The statistical test(s) used AND whether they are one- or two-sided<br>*Only common tests should be described solely by name; describe more complex techniques in the Methods section.*
☒	☐	A description of all covariates tested
☐	☒	A description of any assumptions or corrections, such as tests of normality and adjustment for multiple comparisons
☐	☒	A full description of the statistical parameters including central tendency (e.g. means) or other basic estimates (e.g. regression coefficient) AND variation (e.g. standard deviation) or associated estimates of uncertainty (e.g. confidence intervals)
☐	☒	For null hypothesis testing, the test statistic (e.g. *F*, *t*, *r*) with confidence intervals, effect sizes, degrees of freedom and *P* value noted<br>*Give P values as exact values whenever suitable.*
☒	☐	For Bayesian analysis, information on the choice of priors and Markov chain Monte Carlo settings
☒	☐	For hierarchical and complex designs, identification of the appropriate level for tests and full reporting of outcomes
☒	☐	Estimates of effect sizes (e.g. Cohen's *d*, Pearson's *r*), indicating how they were calculated

Our web collection on statistics for biologists contains articles on many of the points above.

Software and code

Policy information about availability of computer code

Data collection	FACS Diva 8.0<br>Leica LAS X Hardware Configurator<br>MILabs-Acquisition-11.00_RC2<br>Summit 6.3.1<br>Maestro 2.10.0<br>UVWin V6.0.0<br>Promega E6080<br>Gene CHS 2.04<br>StepOne Software PULS
Data analysis	FACS Diva 8.0<br>Summit 6.3.1<br>MILabs Rec 10.16<br>Maestro 2.10.0<br>Leica LAS X Hardware Configurator<br>Microsoft Excel 2016<br>Image J (ij152-win-java8)<br>GraphPad Prism 8.2.1<br>CaseViewer 2.4<br>FlowJo v10

StepOne Software PULS
Imaris Software 9.3.1

For manuscripts utilizing custom algorithms or software that are central to the research but not yet described in published literature, software must be made available to editors and reviewers. We strongly encourage code deposition in a community repository (e.g. GitHub). See the Nature Portfolio guidelines for submitting code & software for further information.

Data

Policy information about availability of data

All manuscripts must include a data availability statement. This statement should provide the following information, where applicable:

- Accession codes, unique identifiers, or web links for publicly available datasets
- A description of any restrictions on data availability
- For clinical datasets or third party data, please ensure that the statement adheres to our policy

All data needed to evaluate the conclusions in the paper are present in the paper and/or the Supplementary Information. The RNA sequencing data of pulmonary fibrosis model mice generated in this study have been deposited in the GEO database under accession code GSE228129 [https://www.ncbi.nlm.nih.gov/geo/query/acc.cgi?acc=GSE228129]. The raw data generated in this study are provided in the Source Data file. The original data and figures used in this study are available in the Figshare database [https://doi.org/10.6084/m9.figshare.23684148].

Research involving human participants, their data, or biological material

Policy information about studies with human participants or human data. See also policy information about sex, gender (identity/presentation), and sexual orientation and race, ethnicity and racism.

Reporting on sex and gender	Two male and two female patients were involved in the present study. The sex has no impacts on the results because only the discarded lung tissues from patients after tumor surgical resection were collected for isolation of lung epithelial cells.
Reporting on race, ethnicity, or other socially relevant groupings	All the involved patients are Chinese. The present study did not involve the socially relevant categorization variables.
Population characteristics	The patients meet the clinical diagnosis of lung tumor, and they must have the clinical needs of surgical resection of tumor.1) Age of 18-60 years old, male or female;2) able to complete the study and all tests;3) meet the criteria for surgical resection of tumors;4) Written informed consent was provided before entering the study, and patients understood that they could withdraw from the study at any time without any loss.
Recruitment	Discarded lung tissues from patients after tumor surgical resection were collected and stored in precooled cell culture medium for subsequent experimental manipulations. Patients were excluded if they met any of the following criteria:1) Participate in other clinical research.2) not voluntarily, or voluntarily but strongly opposed by family members.3) upper or lower respiratory tract infection or related symptoms (including common cold) within 2 weeks. The participated patients were gave informed consent under the auspices of Institutional Review Board of the Second Affiliated Hospital, Zhejiang University School of Medicine.
Ethics oversight	Ethics Committee of the Second Affiliated Hospital, Zhejiang University School of Medicine (Approval number: 0153).

Note that full information on the approval of the study protocol must also be provided in the manuscript.

Field-specific reporting

Please select the one below that is the best fit for your research. If you are not sure, read the appropriate sections before making your selection.

☒ Life sciences ☐ Behavioural & social sciences ☐ Ecological, evolutionary & environmental sciences

For a reference copy of the document with all sections, see nature.com/documents/nr-reporting-summary-flat.pdf

Life sciences study design

All studies must disclose on these points even when the disclosure is negative.

Sample size	No statistic methods were used to predetermine sample sizes. Sample sizes were set based on previous study (PMID: 34586849) with some modifications to meet the current standards for in vivo and in vitro studies. Sample size for each experiment was set at least three to obtain reliable results and was indicated in figure legends. Each sample represents independent biological replicates.
Data exclusions	No data were excluded.
Replication	All experiments were successfully replicated. In vitro cell experiments were repeated for 3 times. For animal studies, a minimum of 3 mice were used. All cell and animal experiments were replicated in at least 3 independent experiments. For IHC or IF experiments, preliminary staining was performed (n=3), then optimised staining performed (minimum n=3). Image analysis was performed at the same time for each experiment.

Randomization	Cell samples were randomly assigned to different groups after cell number counting, keeping the same cell number of each experimental and control group at the beginning. For animal studies, age matched mice were randomly assigned to different treatment groups and control group. All lung samples in each group were harvested after the treatment and were randomly assigned for different determinations.
Blinding	Investigators were blinded to group allocation during the experiments and the data collection and analysis.

Reporting for specific materials, systems and methods

We require information from authors about some types of materials, experimental systems and methods used in many studies. Here, indicate whether each material, system or method listed is relevant to your study. If you are not sure if a list item applies to your research, read the appropriate section before selecting a response.

Materials & experimental systems

n/a	Involved in the study	
☐	☒	Antibodies
☐	☒	Eukaryotic cell lines
☒	☐	Palaeontology and archaeology
☐	☒	Animals and other organisms
☒	☐	Clinical data
☒	☐	Dual use research of concern
☒	☐	Plants

Methods

n/a	Involved in the study	
☒	☐	ChIP-seq
☐	☒	Flow cytometry
☒	☐	MRI-based neuroimaging

Antibodies

Antibodies used

mouse anti-CD31 [clone: 390, FITC] (Biolegend, 102405), 1:200
mouse anti-CD45 [clone: 30-F11, PerCP/Cyanine5.5] (Biolegend, 103132), 1:200
mouse anti-CD326 [clone: G8.8, PE] (Biolegend, 118205), 1:200
goat anti-rabbit IgG (H+L) Secondary Antibody [polyclonal, FITC] (Boster, BA1105), 1:100
rabbit anti-EpCAM (Affinity, DF6311), 1:500
rabbit anti-Ki67 (Boster, PB9026), 1:200
rabbit anti-VIM (Boster, BM4029), 1:100
rabbit anti-α-SMA (Boster, BM3902), 1:100
rabbit anti-collagen, type 1 (Proteintech, 14695-1-AP), 1:500
rabbit anti-LC3B (CST,43566), 1: 1000
rabbit anti-GAPDH (Abcam, ab181602), 1: 10000
goat anti-rabbit IgG (H+L) (Thermo Pierce, 31210), 1:5000
goat anti-mouse IgG (H+L) (Thermo Pierce 31431), 1:5000

Validation

mouse anti-CD31 [clone: 390, FITC] (Biolegend, 102405); https://www.biolegend.com/en-us/products/fitc-anti-mouse-cd31-antibody-377
mouse anti-CD45 [clone: 30-F11, PerCP/Cyanine5.5] (Biolegend, 103132); https://www.biolegend.com/en-us/products/percp-cyanine5-5-anti-mouse-cd45-antibody-4264
mouse anti-CD326 [clone: G8.8, PE] (Biolegend, 118205); https://www.biolegend.com/en-us/products/pe-anti-mouse-cd326-ep-cam-antibody-4726
goat anti-rabbit IgG (H+L) Secondary Antibody [polyclonal, FITC] (Boster, BA1105); https://www.boster.com.cn/index/products/productsDetail?goods_sn=BA1105
rabbit anti-EpCAM [polyclonal] (Affinity, DF6311); https://affbiotech.cn/goods-5113-DF6311-EpCAM_Antibody.html
rabbit anti-Ki67 [polyclonal] (Boster, PB9026); https://www.bosterbio.com/anti-ki67-picoband-trade-antibody-pb9026-boster.html
rabbit anti-VIM [monoclonal] (Boster, BM4029); https://www.boster.com.cn/home/product/anti-vim-antibody_bm4029.html
rabbit anti-α-SMA [monoclonal] (Boster, BM3902); https://www.boster.com.cn/home/product/anti-sma-antibody_bm3902.html
rabbit anti-collagen, type 1 [polyclonal] (Proteintech, 14695-1-AP); https://www.thermofisher.cn/cn/zh/antibody/product/Collagen-Type-I-Antibody-Polyclonal/14695-1-AP
rabbit anti-LC3B [monoclonal] (CST,43566); https://www.cellsignal.cn/products/primary-antibodies/lc3b-e7x4s-xp-rabbit-mab/43566
rabbit anti-GAPDH [monoclonal EPR16891] (Abcam, ab181602); https://www.abcam.cn/products/primary-antibodies/gapdh-antibody-epr16891-loading-control-ab181602.html
goat anti-rabbit IgG (H+L) [polyclonal] (Thermo Pierce, 31210); https://www.thermofisher.cn/cn/zh/antibody/product/Goat-anti-Rabbit-IgG-H-L-Secondary-Antibody-Polyclonal/31210
goat anti-mouse IgG (H+L) [polyclonal] (Thermo Pierce, 31431); https://www.thermofisher.cn/cn/zh/antibody/product/Goat-anti-Mouse-IgG-H-L-Secondary-Antibody-Polyclonal/31431

Eukaryotic cell lines

Policy information about cell lines and Sex and Gender in Research

Cell line source(s)

Mouse alveolar epithelial cells (TC-1) (BFN60805941) were gifted by Prof. Peihua Luo from Zhejiang University, which were purchased from Bluefcell Biotechnology (Shanghai, China).
Human lung epithelial cells (BEAS-2B, ZQ0381) and human lung fibroblast (Hs888Lu, ZQ005) were purchased from ScienCell

	Research Laboratories Inc. (Shanghai, China).<br>Human umbilical vein endothelial cell (HUVEC, KG419) was purchased from KeyGEN Biotech Co., Ltd. (Nanjing, China).
Authentication	Human mesenchymal stem cells (hMSC) isolated from placenta (passages 2 to 6) were analyzed by flow cytometry, which revealed that the cells expressed high levels of CD29 (100%), CD73 (95.3%), CD13 (100%), and CD90 (85.2%), moderate levels of CD105 (69.4%) and CD166 (44.1%), but expressed almost no hematopoietic cell markers such as CD45 (0.1%), CD34 (0.5%), and CD133 (0.2%). CD31 and CD79b were expressed in only 0.2 and 0.4% of the population, respectively.<br>TC-1 cells were authenticated by morphology. BEAS-2B, Hs888Lu, and HUVEC were authenticated by STR profiling.
Mycoplasma contamination	All cells used were not mycoplasma contamination.
Commonly misidentified lines (See ICLAC register)	The study did not involve commonly misidentified lines.

Animals and other research organisms

Policy information about studies involving animals; ARRIVE guidelines recommended for reporting animal research, and Sex and Gender in Research

Laboratory animals	Mice used in this study were all C57BL/6 background. Only male mice with 6-week-old were used in the experiment, with the weight range of 18 g to 22 g. Mice were housed in an animal feeding system of individually ventilated cages (IVC system, ZJ-4, Suzhou Fengshi Laboratory Equipment Co., Ltd. Suzhou, China) under standard laboratory housing conditions (25±1°C, 50% relative humidity and 12 h/12 h dark/light cycle) .
Wild animals	The study did not involve wide animals.
Reporting on sex	Only male C57BL/6 mice were studied in the present study. The sex of mice has no impacts on the findings.
Field-collected samples	The study did not involve animals collected from fields.
Ethics oversight	All animal experimental procedureswere performed with the approval of the Animal Experimental Ethics Committee of Zhejiang University.

Note that full information on the approval of the study protocol must also be provided in the manuscript.

Flow Cytometry

Plots

Confirm that:

☒ The axis labels state the marker and fluorochrome used (e.g. CD4-FITC).

☒ The axis scales are clearly visible. Include numbers along axes only for bottom left plot of group (a 'group' is an analysis of identical markers).

☒ All plots are contour plots with outliers or pseudocolor plots.

☒ A numerical value for number of cells or percentage (with statistics) is provided.

Methodology

Sample preparation	For cell lines, the cells were trypsinisation and washed by PBS for 3 times.<br>For cells from tissues, lung samples were harvested as described above, followed by dissolving in the PBS solution containing 2 mg/mL collagenase and 50 U/mL DNase I for 40 min. The cell suspensions were then centrifuged twice at 2800 rpm with 4 °C, and each time for 5 min to harvest the precipitates. The obtained cell pellets were resuspended in 1% BSA.
Instrument	BD Fortessa
Software	Diva software V8.0
Cell population abundance	Cells were flow sorted, the post-sorted cell population is at least 10000 events.
Gating strategy	The gating strategy is as follows: for distinct GFP-TC-1 population, cell debris was first gated out. Then the single cell population was gated by FSC-H/FSC-W and SSC-H/SSC-W. The FITC positive cell population was regarded as the GFP-TC-1. For isolating alveolar epithelial cells, cell debris was first gated out, followed by the gatiing of CD31-CD45-CD326+ population.

☒ Tick this box to confirm that a figure exemplifying the gating strategy is provided in the Supplementary Information.
